# Frozen Elephant Trunk in Aortic Arch Disease: Different Devices for Different Pathologies

**DOI:** 10.3390/medicina57101090

**Published:** 2021-10-12

**Authors:** Carlo Mariani, Giacomo Murana, Alessandro Leone, Luca Di Marco, Davide Pacini

**Affiliations:** 1 Division of Cardiac Surgery, IRCCS Azienda Ospedaliero-Universitaria di Bologna, Massarenti Street 8, 40138 Bologna, Italy; carlo.mariani5@unibo.it (C.M.); alessandro.leone@aosp.bo.it (A.L.); luca.dimarco@aosp.bo.it (L.D.M.); davide.pacini@unibo.it (D.P.); 2 Department of Experimental, Diagnostic and Specialty Medicine, DIMES, University of Bologna, 40138 Bologna, Italy

**Keywords:** frozen elephant trunk, aortic dissection, aortic aneurysm

## Abstract

The frozen elephant trunk technique (FET) requires the use of a pre-assembled hybrid prosthesis consisting of a standard Dacron vascular portion to replace the aortic arch and a stent graft component, which is placed into the proximal descending thoracic aorta (DTA) anterogradely in the proximal descending thoracic aorta. In Europe, two hybrid prostheses are available: the E-evita Open Plus hybrid stent graft system provided by JOTEC (Hechingen, Germany) and the ThoraflexTM Hybrid (Vascutek, Inchinnan Scotland). Recommendations for use are extensive pathologies of the arch in case of acute and chronic aortic dissection, degenerative aneurysm and intramural hematoma. The FET approach allows the replacement of the whole arch in one stage with the option of direct treatment of the proximal descending thoracic aorta based on the stent component, creating a safe landing zone for further endovascular treatment more distally. The remarkable feature of this technique is the possibility to perform more proximally (from zone 3 to zone 0) the distal anastomosis in to the arch. This allows for an easier distal anastomosis, reduced hypothermic circulatory arrest time and decreased risk of paraplegia (<5%). Early results are promising and according to the most recent series the rate of developing post-operative renal insufficiency ranges from 3 to 10%, the risk of stroke from 3% to 8% and mortality from 8–15%. The aim of the article will be to provide some knowledge about the use and application of FET procedures in different aortic situations.

## 1. Introduction

Complex aortic disease involving the thoraco-abdominal aorta is one of the hardest for cardiac surgeons to handle. The ideal treatment should carry an operative risk within acceptable boundaries and play a key role for subsequent procedures as required. For this reason, the conventional elephant trunk (CET) and even more the frozen elephant trunk (FET) fulfil these requirements for the treatment of complex aortic disease. The elephant trunk technique requires the use of a pre-assembled hybrid prosthesis consisting of a standard Dacron vascular portion to replace the aortic arch and a stent graft (SG) component, which is placed into the proximal descending thoracic aorta anterogradely. The FET approach allows the replacement of the whole arch in one stage with the option of direct treatment of the proximal descending thoracic aorta based on the stent component, creating a safe landing zone for further endovascular treatment more distally.

## 2. Devices

In Europe, two hybrid prostheses are currently available: the E- evita Open Plus hybrid stent graft system provided by JOTEC (Hechingen, Germany) and the ThoraflexTM Hybrid provided by Vascutek (Inchinnan, Scotland).

The more advanced ThoraflexTM Hybrid graft has a quadrifurcated proximal vascular portion (tubular in the E-vita) to facilitate reimplantation of the epiaortic vessels and a more rapid reperfusion of the lower part of the body once the distal anastomosis is completed [1,2].

### 2.1. Thoraflex Hybrid

The Thoraflex hybrid prostheses is composed by a 4-branched arch gelatin-coated woven polyester prosthesis and a self-expanding stent-graft constructed of thin-walled polyester and nitinol ring stents. The stented portion of these prostheses is available in diverse diameters (28–40 mm) and lengths (100 or 150 mm) [1,2,3].

### 2.2. E-Vita Open and E-Vita Open Plus

The E-Vita Open and the E-Vita Open Plus grafts are composed by a proximal dacron prosthesis for the arch and a distal self-expandable stent graft with Z-nitinol stents for the DTA. It can been delivered anterogradely form the arch by an introducer and the vascular portion is invaginated inside the stent graft, after the suture fixation the incorporate arch prosthesis is released from the endovascular portion and used to replace the arch, in case of the Evita open the tubular graft allows the reimplantation of the supra-aortic vessel ‘en block’. The Evita Open stent graft portion is available between 20 and 40 mm ant the SG length is available 130, 150 and 160 mm [4].

### 2.3. Evita Open Neo and Enovia

A novel evolution of the E-Vita stent graft is the E-vita Open NEO; it’s available in three variants: the first one with a stent and an arch graft with a side branch for lower body perfusion; the second one with a stent graft and the arch graft having individual branch grafts for selective anastomosis to the supra-aortic arch vessels; and third ‘Spielvogel type’: projected for a ‘no-arch-touch’ technique with the suture line in Zone 0. A new prototype is the E-Novia: this prosthesis will be suitable for acute type I aortic dissection, Penn B, C, BC, and patients with severe concomitant disease. It will have a covered and non-covered stent-graft portion. The covered stent-graft will be released in the descending thoracic aorta and non- covered stent-graft in the aortic arch (similarly the petticoat technique). The anastomosis would be then performed in Zone 0 and hence it could allow a reducing ischemic times as well as hypothermic circulatory arrest time.

## 3. Surgical Indication and Use of FET

FET technique can be applied extensively for the treatment of several pathologies involving the thoracic aorta.

### 3.1. Acute Aortic Dissection

In acute aortic dissection, the endovascular portion of the FET can be helpful because in case of malperfusion the stenting of the proximal DTA is ideal to re-establish the correct flow in the true lumen and solve malperfusion. Furthermore, the SG expand the true lumen supporting its patency and depressurizing the false lumen. The combination of surgical and endovascular portion, support the surgical anastomosis from the inside; this reduce the risk of development of pseudoaneurysm at this level and distal anastomosis related complication. Distal stent portion also allow a positive remodeling of the downstream aorta and stabilization of the TL in the majority of the cases [5,6,7].

### 3.2. Chronic Aortic Dissection

Residual aortic dissection represents the most frequent indication for the use of the frozen elephant trunk [8].

**Figure 1:**
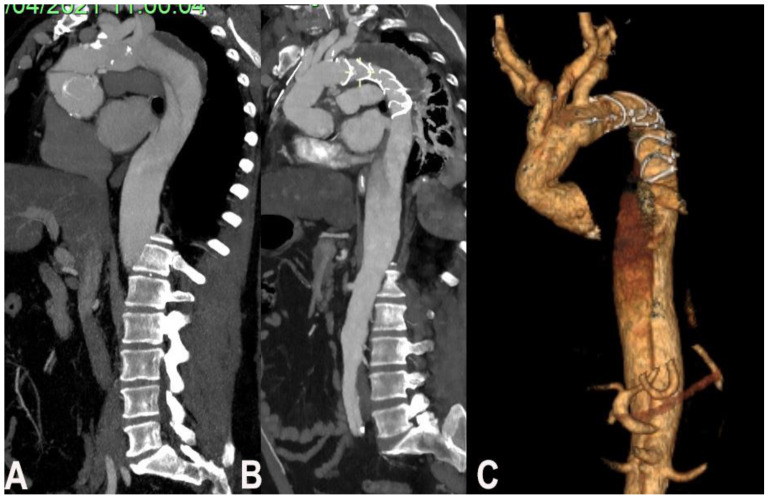
(**A**). Chronic residual aortic dissection, (**B**) post-operative CT scan, (**C**) post-operative CT scan reconstruction.

The possibility to use FET depends on entry tear position and involvement of the thoraco-abdominal aorta including visceral branches. The study of visceral branches should be accurately evaluated, because a selective origin from the false lumen represent a contraindication to FET since the high risk of FL thrombosis and hence malperfusion (Figure 1).

### 3.3. Thoraco-Abdominal Aneurysm

**Figure 2:**
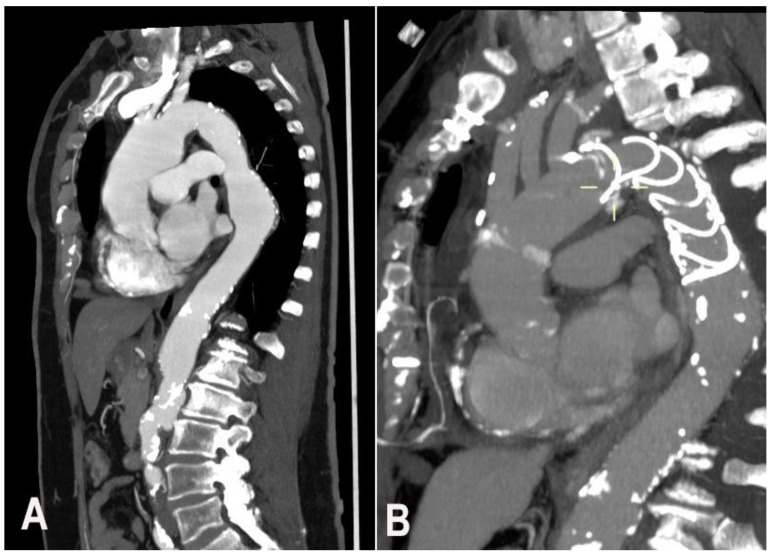
(**A**). Degenerative aneurism of the descending thoracic aorta, (**B**). Post FET CT scan.

In case of complex thoracic aorta aneurysm that would require a double step surgery may qualify for FET technique. The second step in most of the cases is imperative and it can be even surgical even endovascular. In case of severe dilatation and tortuosity of thoracoabdominal (not suitable for a second endovascular stage) a classic elephant trunk is preferred to facilitate the second open stage repair (Figure 2).

### 3.4. Intramural Hematoma

According to guidelines, the treatment of a type A intramural hematoma it’s the same for type A aortic dissection [9]. Therefore, especially in case of aortic arch involvement (i.e., retrograde type B IMH), a more radical treatment with the frozen elephant trunk (FET) it reasonable in order to repair in one stage a large portion of the aorta especially in young patients [10].

### 3.5. Aortic Arch Anomalies

FET technique is also suitable in case of aortic anomalies such us aberrant subclavian artery or in case of vascular rings and fistulae [11,12].

## 4. Surgical Technique—How We Do It in Bologna

Our surgical approach include a full median sternotomy and antegrade selective cerebral perfusion (ASCP) with a flow rate of 12 mL/kg/min and moderate hypothermia as method of brain protection. A central arterial cannulation for cardiopulmonary bypass institution is usually preferred using a direct cannulation of the axillary, innominate artery or carotid artery or through an 8-mm graft interposition. Venous drainage is achieved by cannulation of the right atrium or femoral vein in cases of complex reoperations. In all patients, near-infrared spectroscopy is used to monitor cerebral perfusion. Circulatory arrest was performed at a target nasopharyngeal temperature of 25 °C. Myocardial protection is achieved with the infusion of cold crystalloid cardioplegia via the modified Bretschneider solution (Custodiol; Koehler Chemie, Alsbach-Haenlein, Germany). After arch resection and antegrade cerebral perfusion institution, the proximal descending aorta is prepared using an external Teflon felt fixed with some (usually 4) internal pledgeted U-stitches. In patients with aortic dissection, the false lumen was surgically obliterated at the level of the distal stump. The stent-graft system was then introduced in an antegrade fashion in the descending aorta over the previously positioned stiff guide-wire and deployed. A circumferential anastomosis between the sewing collar and the previously prepared native aorta is performed to ensure the correct sealing. Systemic perfusion is then restored using the side branch of the graft. The supra-aortic vessels are then separately reimplanted. In most patients, proximal repair is performed after the left subclavian artery rerouting in order to reduce the cardiac ischemic time. The distal anastomosis can be localized just beyond the left subclavian artery, between the left subclavian artery and the left carotid artery, or even more proximally. It is clear that more proximal distal anastomoses can be performed more easily, with less risk of left recurrent nerve damage [13,14].

Generally, we size the endovascular portion with an oversizing of 10–20% in case of chronic aneurism or chronic aortic dissection, the stent oversizing is not recommended instead in case of acute aortic dissection due to the high risk of distal stent induced new entry (SINE). Perioperative cerebrospinal fluid drainage (with a target spinal pressure <12 mmHg) is recommended in elective cases where a long stent graft implantation is planned or previous vascular or endovascular surgery of the thoraco-abdominal aorta. After the implantation a mean systemic pressure > of 80 mmHg is strongly recommended [15].

## 5. Review of the Literature and Bologna Experience in Different Aortic Pathologies

Many European centers are increasingly using the FET technique (early results are reported in Table 1). Beckmann et al. [16] analyzed 211 patients underwent FET with the trifurcated Thoraflex Hybrid FET graft: degenerative aneurysms in 68 patients, acute aortic dissections (AD) in 96 patients, and chronic ADs in 47 patients. And, 18% of cases were sternal re-operations. In-hospital mortality was 12% and incidence of re-thoracotomy for bleeding, stroke, permanent paraplegia/paraparesis, prolonged ventilatory support (>96 h), and long-term dialysis were 13%, 18%, 2%, 21%, and 5%, respectively.

Another German center in Essen reported their experience with the straight tubular device in 285 patients: 30-day mortality assessed at 11% with an incidence of SCI of 4% [17].

In the recent report of the E-vita Open international registry accounting for 1165 patients the overall in-hospital mortality rate was 12% and permanent cerebral and spinal cord complications occurred in 5.2% and 3.9% respectively [4].

Okita et al. [18] reported an hospital mortality rate of 2.4% with an incidence of spinal cord injury of 3.5% with the Frozenix device. Similar results were reported in other studies from Japan [19], China [20] and Canada [21].

**Figure 3:**
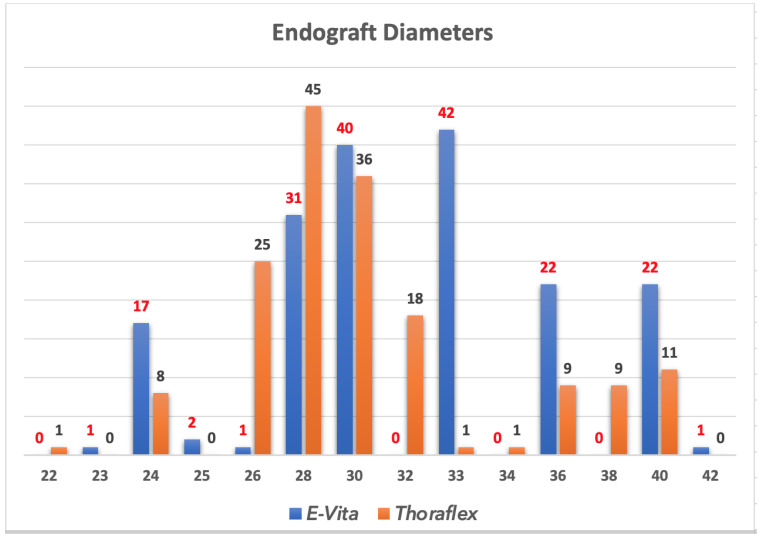
Dimension of the stent graft (mm).

In Bologna, between 2007 and 2021, 343 patients underwent aortic surgery with Frozen Elephant Trunk. Among these, 179 (52.2%) received an E-Vita Open prosthesis while in 165 (47.8%) was used the branched Thoraflex device (Figure 3).

The indications for the FET procedure were: type A chronic dissection in 27 patients (7.9%), residual aortic dissection after treatment of acute type A dissection in 121 patients (35.3%), chronic degenerative aneurysm in 96 patients (28%), and chronic type B aortic dissection in 35 patients (10.2). Furthermore, we used FET in acute setting: 49 patients (14.3%) were operated for acute type A aortic dissection and 15 (4.4%) for acute type B dissection [22].

Mean cardiopulmonary bypass time was 230 ± 69 min, the cerebral protection was performed in all cases with a mean perfusion time of 95 ± 36 min.

During follow-up, 91 (26.5%) patients required endovascular extension at a mean time of 22 months after surgical intervention.

## 6. Comments

The use of FET technique was based initially on custom made prostheses. Subsequently, the availability of different hybrid prostheses has contributed to its increased popularity. The advantage of this approach is to provide distal arch stability and an easy landing zone for later endovascular extension. It also facilitates arch reconstruction because of the possibility to move the distal anastomosis more proximally to the ascending aorta.

Current available hybrid grafts are similar, however, we usually prefer to use the Thoraflex device because of the easy conformation of side branches especially in case of acute aortic dissections. Instead, the E-vita Open stent portion of the graft have a stronger radial force, and this is the reason, we usually prefer to use this graft in chronic degenerative aneurysms.

With both grafts there is the possibility to choose among different sizes and lengths of the stent portion to customize it according to patient anatomy.

The outcomes are very excellent considering the magnitude of the procedure, even do, morbidities remain substantial. The rate of paraplegia ranges from zero to 5%, the risk of developing post-operative renal insufficiency from 3 to 10% and the risk of stroke from 3% to 8% [1,4,14,20].

In the recent results coming from the International E-vita open registry, the authors reported the largest European experience coming from 19 centers and provided complete data on 1165 patients [4]. The data were stratified according to the evolution of FET treatment during time: the 1st period, 2005–2011 was compared with the 2nd period, 2012–2018. In the 2nd period, the number of right axillary artery cannulation increased; the number of direct cannulation of the proximal aorta decreased significantly and the number of other central cannulation techniques, like brachiocephalic trunk, carotid artery, increased. Bilateral selective cerebral perfusion remained the most frequent technique for cerebral protection. More ascending aorta interventions and aortic valve sparing procedures were performed in the 2nd time period. With regard to intraoperative times, visceral ischemia time decreased significantly in the 2nd time period as a positive consequence of an easier graft implantation and simplified FET technique [4].

According to this international experience, the overall 30-day mortality was 12%. In the 2nd time period an improvement was clearly observed in terms of post-operative cerebral complications including stroke (10% vs. 6%) and spinal cord-related symptoms (8% vs. 6%) both also in the incidence of renal injuries (26% vs. 19%) [4].

Despite all the devices improvement and possibility to select among different graft, the most important aspect of the procedure is patient selection.

A pre-operatory angio-TC evaluation is mandatory. The descending thoracic aorta should be carefully evaluated to include the diseased aortic portion within the stent graft and measure the site of distal landing zone. Another important aspect to consider are the exact location of the entry and re-entry tears and the distribution of visceral vessels between the true and false lumens.

In chronic aortic dissections with last of distal re-entries is preferred to use a FET when visceral arteries originate from the true lumen to prevent possible malperfusion syndrome in case of early thrombosis of the false lumen due to stent coverage.

In patients with acute type A aortic dissection a FET is indicated when the entry tear is located in the distal arch or proximal descending aorta, in case if arch rupture or in case of visceral malperfusion due to true lumen compression.

In patients with chronic aortic aneurysm of the arch and descending thoracic aorta, the FET technique can be used as a first approach when further endovascular extensions are expected especially when a ‘‘single-stage’’ procedure can be obtained. However, in very dilated and tortuous thoraco-abdominal aneurysm when a second open stage procedure is necessary a conventional elephant trunk procedure should be strongly considered.

In summary, the most appreciated adjunct of this procedure is represented by the possibility to move more proximal the distal anastomosis in the arch (from zone 3 to zone 0). The advantages are to perform an easier distal anastomosis, reduced hypothermic circulatory arrest time and minimize the risk of post-operative paraplegia.

## Figures and Tables

**Table 1:** Early results of principal retrospective studies.

Author/Year	Device	Patients	Hospital Mortality	SCI
Beckmann et al. 2020 [16]	Thoraflex	211	12%	2%
Tsagakis et al. 2018 [17]	E-Vita	286	11%	4%
Tzagakis et al. 2020 [4]	E-Vita	1165	12%	7%
Okita et al. 2020 [18]	Frozenix	372	2.4%	3.5%
Uchida et al. 2016 [19]	Frozenix	60	5%	6.7%
Qi et al. 2018 [20]	Four-branch prosthetic graft (Boston Scientific, Inc., Boston, MA) with stent-graft	53	0%	NA
Ouzounian et al. 2020 [21]	Thoraflex E-vita	80 5	8%	1.8%

SCI: spinal cord injury.
